# The odor stick identification test for Japanese differentiates Parkinson's disease from multiple system atrophy and progressive supra nuclear palsy

**DOI:** 10.1186/1471-2377-11-157

**Published:** 2011-12-22

**Authors:** Masahiko Suzuki, Masaya Hashimoto, Masayuki Yoshioka, Maiko Murakami, Keiichi Kawasaki, Mitsuyoshi Urashima

**Affiliations:** 1 Department of Neurology, Aoto Hospital, Jikei University School of Medicine, 6-41-2 Aoto Katsushika-ku, Tokyo 125-8506, Japan; 2 Division of Molecular Epidemiology, Jikei University School of Medicine, 3-25-8 Nishi-shimbashi, Minato-ku, Tokyo 105-8461, Japan

## Abstract

**Background:**

Progressive supranuclear palsy (PSP) and parkinsonian variant of multiple system atrophy (MSA-P) are clinically difficult to differentiate from idiopathic Parkinson's disease (PD), particularly in the early stages of the disease. Previous reports indicated that the olfactory function is relatively intact or slightly reduced in patients with PSP and MSA-P, suggesting that the odor stick identification test for Japanese (OSIT-J), which is a short and simple noninvasive test that is potentially useful clinically for detecting early-stage PD in Japan, may be useful in the differential diagnosis of early-stage PD from MSA-P and PSP. There is no information on the sensitivity and specificity of OSIT-J in the diagnosis of parkinsonian syndromes such as PSP and MSA-P.

**Methods:**

We assessed the olfactory function using the OSIT-J test in 94 Japanese patients with idiopathic PD, 15 with MSA-P, 7 with PSP, and 29 age-matched control subjects.

**Results:**

The mean ± SD score of OSIT-J in patients with PD (4.4 ± 2.9) was significantly lower than in patients with MSA-P (8.7 ± 2.2, P < 0.0001), PSP (7.6 ± 2.2, P < 0.0057), and control subjects (10.5 ± 1.3, P < 0.0001). The area under the curve (AUC) of receiver operating characteristic (ROC) to discriminate PD from normal control using OSIT-J scores was 0.97 (95% confidence interval, 0.95-1.00), from MSA-P 0.87 (0.80-0.95), and from PSP 0.81 (0.66-0.96).

**Conclusion:**

The OSIT-J is a potentially useful clinical test not only for detection of olfactory deficit in PD but also for differentiating PD from MSA-P and PSP.

## Background

Olfactory dysfunction is recognized as a non-motor symptom in idiopathic Parkinson's disease (PD), and is a maker for preclinical diagnosis of PD based on the appearance of pathological changes in the olfactory system before the development of motor symptoms [1]. A recent study indicated that the early appearance of impaired olfaction prior to other clinical features of PD could be a useful screening tool to detect those at high risk for the development of PD in later life [2]. In fact, several Japanese researchers employ the 12-odorant test, the Odor Stick Identification Test for Japanese (OSIT-J) in the clinical testing of PD [3-5] and have reported their findings of dysosmia and that it did not correlate with motor function, disease duration, or medication, indicating that OSIT-J scores are independent of all other measures in PD [3,4]. In addition to the practice in Japan, the results of the University of Pennsylvania 12 smell identification test (UPSIT), which is also a smell identification test [6-8], indicate that OSIT-J is a short and simple noninvasive test that is potentially useful clinically for detecting early-stage PD.

The diagnosis of PD is based on clinical criteria, the accuracy of such diagnosis even in patients with chronic condition is 90% at best [9]. Progressive supranuclear palsy (PSP) and parkinsonian variant of multiple system atrophy (MSA-P) are neurodegenerative disorders that are clinically difficult to differentiate from idiopathic PD, particularly in the early stages of the disease, when the typical clinical signs are not clearly evident [10]. Previous reports indicated that the olfactory function is relatively intact or slightly reduced in patients with PSP and MSA-P [11-15], suggesting that OSIT-J may be useful in the differential diagnosis of early-stage PD from MSA-P and PSP [15,16]. To our knowledge, there is no information on the sensitivity and specificity of OSIT-J in the diagnosis of parkinsonian syndromes such as PSP and MSA-P. The aim of the present study was to evaluate the OSIT-J in patients with idiopathic PD, MSA-P, and PSP.

## Methods

### Patients and Control Subjects

From October 2008 through March 2011, consecutive nondemented patients underwent OSIT-J as part of the initial clinical evaluation of parkinsonism, either as inpatients or outpatients at the Department of Neurology, The Jikei University Aoto Hospital. The clinical diagnosis of PD, MSA-P, PSP was determined by three authors (MS, MY, MH, with more than 10-year experience in movement disorders) according to established consensus criteria. The patients were included in the study only if they fulfilled the criteria for PD [17], MSA-P [18], and PSP [19]. The controls were partners of the patients or subjects with normal neurological examination free of central nervous system (CNS) diseases. Seven patients with PSP (4 men, mean age 70.6 years, mean disease duration 2.4 years), 15 patients with MSA-P (9 men, 67.3 years, 2.1 years), 94 patients with PD (47 men, 68.6 years, 5.1 years) and 29 age-matched healthy subjects (9 men, 66.1 years) were included in this study. All patients were evaluated using the Unified Parkinson's Disease Rating Scale (UPDRS) [20] and Hoehn and Yahr (HY) stage [21] for estimation of disease severity. We excluded patients with Mini Mental State Examination (MMSE) score of less than 24, because it was unlikely that they could adequately comprehend and respond to the odor. The following patients were also excluded from the study: patients with 1) history of stroke (PD; n = 1), epilepsy (PD; n = 1), or psychiatric illness (PD; n = 1), 2) missing clinical data (PD; n = 3, MSA-P; n = 1), and 3) obvious medical complications that could lower the accuracy of clinical diagnosis of PD (n = 3). Two cases with autosomal recessive juvenile parkinsonism [22] was also excluded. The enrolled subjects were free from other conditions that can affect olfactory function such as smoking, usage of certain medications, history of nasal surgery, pulmonary disease, hormonal disorders, perennial allergies, or abuse of drugs or alcohol.

Informed consent was obtained from all participants following a full explanation of the study. As stated in the Introduction, the OSIT-J test is used routinely in clinical practice in Japan and in our hospital. Accordingly, we explained to all participants that the collected clinical data could be used in the future for research purpose but all results will be anonymous. The study was approved by the ethics committee of Jikei university.

### Odor Stick Identification Test for Japanese (OSIT-J)

OSIT-J (Daiichi Yakuhin, Co., Tokyo, Japan) comprises 12 different odorants familiar to the Japanese population [23,24]: rose, condensed milk, Japanese orange, curry, roasted garlic, fermented beans/sweaty socks, cooking gas, menthol, India ink, wood, and Japanese cypress (hinoki) [3,4]. These odors were chosen from clusters representing Japanese daily life and are familiar to the Japanese population, and each odorant was selected from the essential oils, pure chemicals, or mixed odorants produced by Takasago International Corporation (Tokyo). For each odorant, the subject is presented with a card showing four names of odors and is asked to select the odor presented. The total number of correct answers for the 12 odorants presented is the OSIT-J score [3]. Thus, the score of the OSIT-J ranges from 0 to 12. Each odorant was enclosed in melamine resin microcapsules, which were mixed into an odorless solid cream and then shaped like a lipstick. The examiner painted each odor stick in a 2-cm circle on thin paraffin paper, folded the paper in half, rubbed it to grind the microcapsules, and then passed it to the participant. The participant opened the paraffin paper and sniffed it. Participants were directed to avoid eating 30 minutes prior to the examination. The order in which the odorants were presented was randomized.

### Statistical Analysis

Values were expressed as mean ± standard deviation (SD). A *P *value less than 0.05 was considered statistically significant. The chi-square test was used to compare frequencies between groups. The Kruskal-Wallis test was used to compare continuous variables with skewed data distribution. Receiver operating characteristic (ROC) curves were drawn and the area under the curve (AUC) was computed to discriminate PD from the normal control, MSA-P and PSP with 95% confidence interval (95%CI), 0.95 to 1.00. The association between MMSE and OSIT-J scores was evaluated with Spearman's test. All analyses were performed using the STATA 11.0 software (STATA Corporation, College Station, TX).

## Results

**Figure 1:**
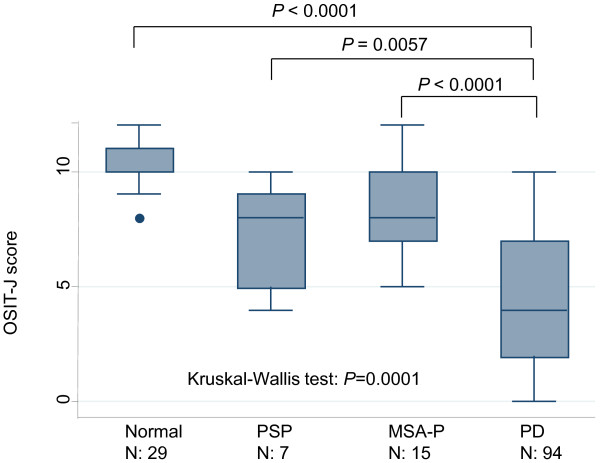
**Box-and-whisker plots of Odor Stick Identification Test for Japanese (OSIT-J) score in normal subjects and patients with Parkinson's disease (PD), parkinsonian variant of multiple system atrophy (MSA-P), and progressive supranuclear palsy (PSP)**. In these plots, lines within the boxes represent median values; the upper and lower lines of the boxes represent the 25th and 75th percentiles, respectively; and the upper and lower bars outside the boxes represent the 90th and 10th percentiles, respectively. The circles represent outlier values.

The disease duration was longer in PD than in MSA-P and PSP, whereas the HY stage was lower in PD than in MSA-P and PSP (Table 1). The OSIT-J scores of PD, MSA-P, PSP, and control subjects are presented in Figure 1. Based on the score, the number of individuals considered to have hyposmia according to the background disease was 76 in PD, 4 in MSA-P, 2 in PSP, and none in the control. The OSIT-J score was 4.4 ± 2.9 in patients with PD, 8.7 ± 2.2 in patients with MSA-P, 7.6 ± 2.2 in patients with PSP, and 10.5 ± 1.3 in the control. The mean OSIT-J score of patients with PD was significantly lower than those with MSA-P (P < 0.0001) and control subjects (P < 0.0001), and marginally lower than patients with PSP (P < 0.0057) (Figure 1).

**Table 1:** Comparison of the four groups of subjects enrolled in the present study.

	control	PD	PD (disease duration ≤ 3 years)	MSA-P	PSP	p value
n	29	94	36	15	7	N/A
Age^1 ^(years)	66.1 ± 8.8	68.6 ± 9.7	70.1 ± 8.6	67.3 ± 9.2	70.6 ± 9.9	ns
Males/females	9/20	47/47	19/16	9/6	4/3	N/A
Disease duration^2 ^(years)	N/A	5.1 ± 4.4	1.5 ± 1.1	2.1 ± 1.7	2.4 ± 2.0	ns
HY stage^2^	N/A	2.3 ± 0.8	2.1 ± 0.7	3.0 ± 0.9*	3.1 ± 0.8*	0.0003
MMSE^1^	29.6 ± 0.7	28.4 ± 1.9	28.6 ± 1.5	28.5 ± 1.9	28.6 ± 1.5	ns
UPDRS (total)^2^	N/A	36.9 ± 17.2	34.7 ± 16.4	34.8 ± 15.6	48.2 ± 18.1	ns
UPDRS (part III)^2^	N/A	23.1 ± 11.5	22.4 ± 11.3	22.8 ± 13.1	26.2 ± 9.8	ns

Data are mean ± SD, ^1^Kruskal-Wallis equality-of-populations rank test, ^2^Mann-Whitney U test for data of PD, MSA-P and PSP groups. N/A, not applicable; ns, not significant; PD, Parkinson's disease; MSA-P, parkinsonian variant of multiple system atrophy; PSP, progressive supranuclear palsy; HY stage, Hoehn Yahr stage; MMSE, mini mental state examination; UPDRS, Unified Parkinson's Disease Rating Scale.

**Figure 2:**
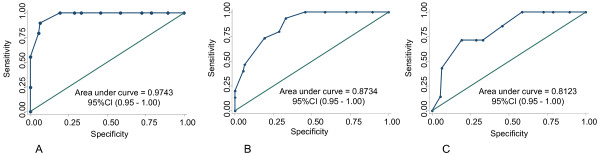
**Receiver operating characteristic (ROC) curves**. ROC curves show the relationship between sensitivity and specificity for OSIT-J score in PD vs normal control subjects (A), in PD vs MSA-P (B), and in PD vs PSP (C).

The area under the ROC curve (AUC) that discriminated PD from normal control based on the OSIT-J score was 0.97 (95%CI, 0.95-1.00) (Figure 2A), PD from MSA-P was 0.87 (0.80-0.95) (Figure 2B), and PD from PSP was 0.81 (0.66-0.96) (Figure 2C). The use of an OSIT-J scale cutoff of 7 or less, discriminated PD from normal control with 81% sensitivity and 100% specificity (Table 2), PD from MSA-P with 81% sensitivity and 73% specificity, and PD from PSP with 81% sensitivity and 71% specificity. Because the control group comprised mainly females while the PD group was mainly males, we analyzed the data of sex-matched control-PD. The results were similar to those of the entire group: males only; sensitivity: 89%, specificity: 100%, females only; sensitivity: 72%, specificity: 100%.

**Table 2:** Discriminate analysis for Odor Stick Identification Test for Japanese (OSIT-J) in parkinsonian disorders.

	OSIT-J score (*≤ *7	OSIT-J score (≥ 8)	Sensitivity (95%CI)	Specificity (%)	Positive predictive value	Negative predictive value
PD/Control	76/0	18/29	81 (71-88)	100 (88-100)	100 (95-100)	62 (46-75)
Males	42/0	5/9	89 (77-96)	100 (66-100)	100 (92-100)	64 (35-87)
Females	34/0	13/20	72 (57-84)	100 (83-100)	100 (90-100)	61 (42-77)
PD/MSA-P	76/4	18/11	81 (71-88)	73 (45-92)	95 (88-99)	38 (21-58)
PD/PSP	76/2	18/5	81 (71-88)	71 (29-96)	97 (91-100)	78 (56-93)

We also analyzed the data taking into consideration the PD disease duration. To this effect, we included data of PD patients with 3yashsoyo h disease (n = 36). The clinical profile of these patients is shown in Table 1. Table 3 shows the results of analyses of the four groups using the data of PD patients with disease history ≤ 3 years. Like the analysis of the entire PD group, the OSIT-J discriminated PD from normal control with 78% sensitivity and 100% specificity (Table 3), PD from MSA-P with 78% sensitivity and 73% specificity, and PD from PSP with 78% sensitivity and 71% specificity.

**Table 3:** Discriminate analysis for Odor Stick Identification Test for Japanese (OSIT-J) in parkinsonian disorders.

	OSIT-J score (7)	OSIT-J score (8)	Sensitivity (95%CI)	Specificity (%)	Positive predictive value	Negative predictive value
PD/Control	28/0	8/29	78 (61-90)	100 (88-100)	100 (88-100)	78 (62-90)
PD/MSA-P	28/4	8/11	78 (61-90)	73 (45-92)	88 (71-96)	58 (33-80)
PD/PSP	28/2	8/5	78 (61-90)	71 (29-96)	93 (78-99)	38 (14-68)

This analysis included data of only 36 patients with Parkinson disease who had ≤ 3-year history of PD.

Finally, the OSIT-J scores in patients with PD, MSA-P, and PSP, did not correlate with age, disease duration, and disease severity including UPDRS scores. However, in 94 patients with PD, there was a positive correlation between the MMSE scores and the OSIT-J scores (P = 0.027).

## Discussion

The present results suggest that OSIT-J is a simple-to-measure and sensitive marker for early-stage idiopathic PD. This conclusion is based on the significantly lower OSIT-J score in patients with PD than the control and its high sensitivity and specificity in the diagnosis. The present results are in agreement with those of previous studies, which showed an OSIT-J score of 4.1-4.8 in patients with PD [3,5,25]. To differentiate PD from the controls, we used cutoff values for OSIT-J of 7, which yielded sensitivity of 81% and specificity of 100% (Table 2). Marked olfactory dysfunction occurs in PD at the earliest stage of the illness, affecting between 70 and 100% of patients and includes impairment in detection threshold, identification, and discrimination [5]. SPECT studies have shown that olfactory test scores correlated with the levels of dopamine transporter within the striatum of the brain of patients with early-stage PD [26]. On the other hand, another study showed that at 4 years from baseline, 7% of individuals with idiopathic olfactory loss exhibited clinical symptoms of PD, and 13% of patients presented with abnormalities of the motor system relevant to PD [2]. Since dysosmia develops before the appearance of symptoms related to motor dysfunction, such impairment could be useful in the detection of not only the early stages of PD but also the premotor phase of PD [5]. Another recent study also suggested that olfactory dysfunction in PD is associated with both cardiac sympathetic and parasympathetic dysfunction as well as with vascular sympathetic dysfunction after adjustment for age, disease duration, motor impairment, and dopaminergic medication. As non-motor symptoms, olfactory and autonomic network dysfunctions appear to be closely related in PD [25]. Since most PD patients appear to become anosmic during later stages of the disease, it becomes clear that the correlation between the duration of PD and olfactory function is only relevant in the relatively early stages of the disease. In turn, this also indicates that smell sensation deficit is an early marker of PD. Based on the neuropathological staging system for PD developed by Braak et al. [1], Lewy body formation in the olfactory bulb and anterior olfactory nucleus precedes the neuronal degeneration in the substantia nigra, suggesting that the olfactory system may represent one of the induction sites of the neuropathological process of PD. This concept is, in general, in agreement with clinical studies that have found olfactory dysfunction to be independent of motor status in PD [27].

Olfaction is markedly impaired in patients with PD. This deficit contrasts with reports of preserved or only mildly reduced olfaction in patients with atypical parkinsonism. To our knowledge, the sensitivity and specificity of olfactory function tests in the diagnosis of parkinsonian syndromes has not been studied in Japan. In addition, there is virtually no information on olfactory function in patients with MSA and PSP. Using the University of Pennsylvania Smell Identification Test (UPSIT) with a test score ranging from 0 to 40, Wenning et al. [15] studied olfactory function in patients with PD (n = 118), MSA (n = 29) and PSP (n = 15), as well as 123 healthy control subjects. The UPSIT scores showed marked impairment in the PD group, in contrast to the mild impairment in MSA patients and normal olfaction in PSP patients [15]. These results demonstrated a differential impairment/preservation of olfactory function in distinct parkinsonian syndromes and that the UPSIT might have some value as a diagnostic marker. Thus, preserved or mild impairment of olfactory function in a parkinsonian patient is more likely to be related to atypical parkinsonism such as MSA and PSP, whereas marked reduction in the UPSIT score is more suggestive of PD. Müller et al. [28] also found profound impairment of olfactory function in almost all patients with PD. Consistent with the results of Wenning et al., they also found evidence for olfactory loss in MSA, but little or no olfactory loss in PSP patients. Several studies have also suggested the lack of correlation between olfactory loss with duration or severity of the disease [27,29,30], which was confirmed by the present study. Studies that examined the correlation between pathological changes in the brain and results of olfactory tests suggested that olfactory dysfunction in MSA could be related to glial cytoplasmic inclusions in the olfactory bulb [31]. Another study examined the brain of patients with PSP at postmortem and identified neurofibrillary tangles and tau accumulation in the rhinencephalon, although only three of their patients had hyposmia [13]. The same study correlated smell test scores with the results of bedside cognitive and motor function tests. Their results confirmed that the olfactory function of patients with PSP is significantly better than that of patients with PD, but in contrast to the two previous publications, it also showed that it was significantly reduced compared with the control after adjustment for age and gender. Our small number of PSP also showed reduced OSIT-J score, though it was not significantly different from the control. It is likely that the difference in the results of the studies is due to differences in the sample size and also certain methodological differences, such as the inclusion of patients with PSP who presented with cognitive impairment.

Admittedly, this study has certain limitations. First, the clinically-based diagnosis was not confirmed pathologically in any of the examined subjects. Second, the number of patients of the PSP group was too small for meaningful conclusions. Third, patients with MSA-P and PSP may have had undiagnosed subclinical or idiopathic rapid-eye-movement sleep behavior disorder [5,32], which could affect the results. Further large scale and longitudinal studies should be conducted to examine the diagnostic utility of OSIT-J in differentiating patients with parkinsonian disorders.

## Conclusion

The present study demonstrated marked impairment of the smell sensation in Japanese PD patients, as tested by a simple, inexpensive and noninvasive OSIT-J test. The OSIT-J could be clinically useful not only for detection of olfactory dysfunction in PD but also for differentiating PD from MSA-P and PSP.

## Competing interests

The authors declare that they have no competing interests.

## Authors' contributions

MS participated in the conceptualization and design of the study and drafted the manuscript. MH, MY, KK and MM conceived of the study, and participated in its design and coordination. MU participated in interpretation of data and performed the statistical analysis. All authors have read and approved the final manuscript.

## Pre-publication history

The pre-publication history for this paper can be accessed here:

http://www.biomedcentral.com/1471-2377/11/157/prepub

## References

[B1] Braak H Rub U Gai WP Del Tredici K Idiopathic Parkinson's disease: possible routes by which vulnerable neuronal types may be subject to neuroinvasion by an unknown pathogen J Neural Transm 2003 110 517 536 10.1007/s00702-002-0808-2 12721813

[B2] Haehner A Hummel T Hummel C Sommer U Junghanns S Reichmann H Olfactory loss may be a first sign of idiopathic Parkinson's disease Mov Disord 2007 22 839 842 10.1002/mds.21413 17357143

[B3] Iijima M Kobayakawa T Saito S Osawa M Tsutsumi Y Hashimoto S Iwata M Smell identification in Japanese Parkinson's disease patients: using the odor stick identification test for Japanese subjects Intern Med 2008 47 1887 1892 10.2169/internalmedicine.47.1345 18981632

[B4] Iijima M Osawa M Momose M Kobayakawa T Saito S Iwata M Uchiyama S Cardiac sympathetic degeneration correlates with olfactory function in Parkinson's disease Mov Disord 2010 25 1143 1149 10.1002/mds.23001 20131383

[B5] Miyamoto T Miyamoto M Iwanami M Suzuki K Inoue Y Hirata K Odor identification test as an indicator of idiopathic REM sleep behavior disorder Mov Disord 2009 24 268 273 10.1002/mds.22361 18972547

[B6] Katzenschlager R Zijlmans J Evans A Watt H Lees AJ Olfactory function distinguishes vascular parkinsonism from Parkinson's disease J Neurol Neurosurg Psychiatry 2004 75 1749 1752 10.1136/jnnp.2003.035287 15548497 PMC1738844

[B7] Ondo WG Lai D Olfaction testing in patients with tremor-dominant Parkinson's disease: is this a distinct condition? Mov Disord 2005 20 471 475 10.1002/mds.20365 15597336

[B8] Silveira-Moriyama L Carvalho Mde J Katzenschlager R Petrie A Ranvaud R Barbosa ER Lees AJ The use of smell identification tests in the diagnosis of Parkinson's disease in Brazil Mov Disord 2008 23 2328 2334 10.1002/mds.22241 18785265

[B9] Hughes AJ Daniel SE Lees AJ Improved accuracy of clinical diagnosis of Lewy body Parkinson's disease Neurology 2001 57 1497 1499 11673599 10.1212/wnl.57.8.1497

[B10] Poewe W Wenning G The differential diagnosis of Parkinson's disease Eur J Neurol 2002 9 Suppl 3 23 30 12464118 10.1046/j.1468-1331.9.s3.3.x

[B11] Goldstein DS Holmes C Bentho O Sato T Moak J Sharabi Y Imrich R Conant S Eldadah BA Biomarkers to detect central dopamine deficiency and distinguish Parkinson disease from multiple system atrophy Parkinsonism Relat Disord 2008 14 600 607 10.1016/j.parkreldis.2008.01.010 18325818 PMC2650101

[B12] Goldstein DS Sewell L Olfactory dysfunction in pure autonomic failure: Implications for the pathogenesis of Lewy body diseases Parkinsonism Relat Disord 2009 15 516 520 10.1016/j.parkreldis.2008.12.009 19201246 PMC4164391

[B13] Silveira-Moriyama L Hughes G Church A Ayling H Williams DR Petrie A Holton J Revesz T Kingsbury A Morris HR Hyposmia in progressive supranuclear palsy Mov Disord 2010 25 570 577 10.1002/mds.22688 20209627

[B14] Silveira-Moriyama L Mathias C Mason L Best C Quinn NP Lees AJ Hyposmia in pure autonomic failure Neurology 2009 72 1677 1681 10.1212/WNL.0b013e3181a55fd2 19433741

[B15] Wenning GK Shephard B Hawkes C Petruckevitch A Lees A Quinn N Olfactory function in atypical parkinsonian syndromes Acta Neurol Scand 1995 91 247 250 7625148 10.1111/j.1600-0404.1995.tb06998.x

[B16] Doty RL Golbe LI McKeown DA Stern MB Lehrach CM Crawford D Olfactory testing differentiates between progressive supranuclear palsy and idiopathic Parkinson's disease Neurology 1993 43 962 965 8492953 10.1212/wnl.43.5.962

[B17] Gelb DJ Oliver E Gilman S Diagnostic criteria for Parkinson disease Arch Neurol 1999 56 33 39 10.1001/archneur.56.1.33 9923759

[B18] Gilman S Low PA Quinn N Albanese A Ben-Shlomo Y Fowler CJ Kaufmann H Klockgether T Lang AE Lantos PL Consensus statement on the diagnosis of multiple system atrophy J Neurol Sci 1999 163 94 98 10.1016/S0022-510X(98)00304-9 10223419

[B19] Litvan I Agid Y Calne D Campbell G Dubois B Duvoisin RC Goetz CG Golbe LI Grafman J Growdon JH Clinical research criteria for the diagnosis of progressive supranuclear palsy (Steele-Richardson-Olszewski syndrome): report of the NINDS-SPSP international workshop Neurology 1996 47 1 9 8710059 10.1212/wnl.47.1.1

[B20] Fahn S Elton RL members Up Fahn S, Marsden CD, Goldstein M, Calne DB Unified Parkinsons Disease Rating Scale Recent developments in Parkinsons diseaseFlorham Park 1987 2 NJ: Macmillan Healthcare Information 153 163

[B21] Hoehn MM Yahr MD Parkinsonism: onset, progression and mortality Neurology 1967 17 427 442 6067254 10.1212/wnl.17.5.427

[B22] Suzuki M Hattori N Orimo S Fukumitsu N Abo M Kono Y Sengoku R Kurita A Honda H Inoue K Preserved myocardial [123I]metaiodobenzylguanidine uptake in autosomal recessive juvenile parkinsonism: first case report Mov Disord 2005 20 634 636 10.1002/mds.20384 15704207

[B23] Kobayashi M Saito S Kobayakawa T Deguchi Y Costanzo RM Cross-cultural comparison of data using the odor stick identification test for Japanese (OSIT-J) Chem Senses 2006 31 335 342 10.1093/chemse/bjj037 16495437

[B24] Saito S Ayabe-Kanamura S Takashima Y Gotow N Naito N Nozawa T Mise M Deguchi Y Kobayakawa T Development of a smell identification test using a novel stick-type odor presentation kit Chem Senses 2006 31 379 391 10.1093/chemse/bjj042 16527871

[B25] Oka H Toyoda C Yogo M Mochio S Olfactory dysfunction and cardiovascular dysautonomia in Parkinson's disease J Neurol 2010 257 969 976 10.1007/s00415-009-5447-1 20119648

[B26] Siderowf A Newberg A Chou KL Lloyd M Colcher A Hurtig HI Stern MB Doty RL Mozley PD Wintering N [99mTc]TRODAT-1 SPECT imaging correlates with odor identification in early Parkinson disease Neurology 2005 64 1716 1720 10.1212/01.WNL.0000161874.52302.5D 15911797

[B27] Doty RL Deems DA Stellar S Olfactory dysfunction in parkinsonism: a general deficit unrelated to neurologic signs, disease stage, or disease duration Neurology 1988 38 1237 1244 3399075 10.1212/wnl.38.8.1237

[B28] Müller A Reichmann H Livermore A Hummel T Olfactory function in idiopathic Parkinson's disease (IPD): results from cross-sectional studies in IPD patients and long-term follow-up of de-novo IPD patients J Neural Transm 2002 109 805 811 10.1007/s007020200067 12111470

[B29] Hawkes CH Shephard BC Daniel SE Olfactory dysfunction in Parkinson's disease J Neurol Neurosurg Psychiatry 1997 62 436 446 10.1136/jnnp.62.5.436 9153598 PMC486843

[B30] Stern MB Doty RL Dotti M Corcoran P Crawford D McKeown DA Adler C Gollomp S Hurtig H Olfactory function in Parkinson's disease subtypes Neurology 1994 44 266 268 8309571 10.1212/wnl.44.2.266

[B31] Kovacs T Papp MI Cairns NJ Khan MN Lantos PL Olfactory bulb in multiple system atrophy Mov Disord 2003 18 938 942 10.1002/mds.10466 12889086

[B32] Miyamoto T Miyamoto M Iwanami M Hirata K Kobayashi M Nakamura M Inoue Y Olfactory dysfunction in idiopathic REM sleep behavior disorder Sleep Med 2010 11 458 461 10.1016/j.sleep.2009.09.013 20378403

